# The Feature Extraction Based on Texture Image Information for Emotion Sensing in Speech

**DOI:** 10.3390/s140916692

**Published:** 2014-09-09

**Authors:** Kun-Ching Wang

**Affiliations:** Department of Information Technology & Communication, Shih Chien University, 200 University Road, Neimen, Kaohsiung 84550, Taiwan; E-Mail: kunching@mail.kh.usc.edu.tw; Tel.: +886-076-678-888-5723; Fax: +886-076-678-888-4332

**Keywords:** emotional feature extraction, emotion sensing, spectrogram, texture image information

## Abstract

In this paper, we present a novel texture image feature for Emotion Sensing in Speech (ESS). This idea is based on the fact that the texture images carry emotion-related information. The feature extraction is derived from time-frequency representation of spectrogram images. First, we transform the spectrogram as a recognizable image. Next, we use a cubic curve to enhance the image contrast. Then, the texture image information (TII) derived from the spectrogram image can be extracted by using Laws' masks to characterize emotional state. In order to evaluate the effectiveness of the proposed emotion recognition in different languages, we use two open emotional databases including the Berlin Emotional Speech Database (EMO-DB) and eNTERFACE corpus and one self-recorded database (KHUSC-EmoDB), to evaluate the performance cross-corpora. The results of the proposed ESS system are presented using support vector machine (SVM) as a classifier. Experimental results show that the proposed TII-based feature extraction inspired by visual perception can provide significant classification for ESS systems. The two-dimensional (2-D) TII feature can provide the discrimination between different emotions in visual expressions except for the conveyance pitch and formant tracks. In addition, the de-noising in 2-D images can be more easily completed than de-noising in 1-D speech.

## 1. Introduction

Human emotion recognition can facilitate emotional information from speech, facial expressions, gestures, and so forth. Recently, emotion recognition methods have fused both speech and visual data to recognize human emotional states. It is desirable to improve the recognition accuracy for audiovisual-based methods while comparing the results with emotion sensing in speech. In [1–3], some feature extraction methods have been provided to increase the accuracy of facial expression recognition. However, there is a tradeoff between accuracy and computing power for recognizing human emotional states from audiovisual signals. In fact, speech can provide a most natural and fundamental interface for human-computer interaction (HCI). With the exponential growth in available computer power and the significant progress in speech technologies, emotion sensing in speech (ESS) systems has important roles in HCI. ESS has several potential applications, such as the interfaces with robots [4–6], call center environments [7], and enhancement of speech and speaker recognition performance [8].

In general, ESS is a computational task consisting of two major parts: feature extraction and emotion machine classification. In fact, the emotional feature extraction part is a crucial issue in any ESS system and is emphasized in this paper. The extracted features must carry sufficient information to represent the emotional states of a speaker. From the reported findings on speech features, most works adopt prosodic features [9–12]. For example, Schuller *et al.* [9] utilized 20 pitches and energy-related features to recognize seven discrete emotions. In [10], the authors used pitch, formant, intensity, speech rate and energy related features to classify neutrality, anger, laughter and surprise. In [11], fundamental frequency, energy and audible duration features were extracted to recognize sadness, boredom, happiness and anger in a corpus recorded by eight professional actors. In [12], the prosodic features, derived from pitch, loudness, duration and quality features were extracted to recognize a 400-utterance database. Those features are regarded as pitch-related features, energy-related features and speaking rate ones. Other features mentioned in the literature are spectral features [13–15]. In [13], the Mel-frequency predictive cepstral coefficients (MFCCs) were selected with pitch, log energy, formant and band energies to perform in a SONY AIBO database. In [14], various speech features, namely, energy, pitch, zero crossing, phonetic rate, linear predictive cepstral coefficients (LPCCs) and their derivatives, were tested and combined with MFCCs. In [15], the short time log frequency power coefficients along with MFCCs were adopted as emotion speech features to recognize six emotions in a 60-utterance corpus. In general, these spectral features are LPCCs-related or MFCCs-related features.

According to the above statement, the conventional feature parameters are usually based on one-dimensional (1-D) information. A two-dimensional (2-D) Gabor filter bank was applied to mel-spectrograms in [16,17]. The resulting outputs of the Gabor filters were concatenated into two-dimensional vectors and used as features in the speech recognition experiments. In [18], a similar method was applied in speech discrimination and enhancement. In recent studies [19–21], a 2-D Gabor filter bank was described for decomposing localized patches of spectrograms into components representing speech harmonistic, formants, vertical onsets/offsets, noise and overlapping simultaneous speakers.

For a few years now, a large number of low-level descriptors (LLD) and functional have promoted the extraction of very large feature vectors (brute-force extraction), and up to many thousands of features are obtained either by analytical feature generation [22–24] or, in a few studies, by evolutionary generation. Such brute-forcing also often includes hierarchical functional application (e.g., mean of maxima) to better cope with statistical outliers. In [25], Schuller's group used large scale brute-force feature sets for emotion recognition. In Escalona Mena's work [26], the author proposed a signal feature extraction using acoustic low-level descriptors. The average accuracy is above 82% for seven-class tasks.

Compared with the conventional feature extraction, we present in this paper a novel texture image feature for ESS systems. This idea is based on the fact that the texture images carry emotion-related information. The feature extraction is derived from time-frequency representation of spectrogram images. First, we transform spectrograms as recognizable images. Next, we use a cubic curve to enhance the contrast of these speech spectrogram images. Then, the texture image information (TII) derived from the spectrogram images can be extracted by using Laws' masks to characterize emotional states. In addition, large scale brute-force feature sets are utilized with the proposed TII features. In order to evaluate the effectiveness of the proposed emotion recognition in different languages, we used two open emotional databases, including the Berlin Emotional Speech Database (EMO-DB), and the eNTERFACE corpus and one self-recorded database (KHUSC-EmoDB), to evaluate the cross-corpora performance. The results of the proposed TII-base ESS system are presented using support vector machine (SVM) as a classifier. It is found that the large-scale brute-force feature sets also improve the average accuracy of emotion recognition, regardless of the need for more computational time.

The remainder of this paper is organized as follows: Section 2 introduces the emotion speech database including two open databases (EMO-DB and eNTERFACE) and one self-recorded database (KHUSC-EmoDB). Section 3 defines the framework of the proposed ESS system. Next, texture image information for the paper's main motivation, spectrogram image calculation for transforming 1-D speech signals into 2-D images, a cubic curve method for image enhancement, Laws' mask for feature definition, and the SVM-based machine for classification are separately described in detail. In addition, the utilized large-scale brute-force feature sets together with the proposed TII features are also presented. The experiments and results are discussed in Section 4. Finally, Section 5 provides the discussion and conclusions.

## 2. Emotional Speech Database

To demonstrate effectiveness of the proposed TII-based feature extraction applied to ESS systems, we carried out experiments on three emotional datasets: EMO-DB, eNTERFACE and KHUSC-EmoDB. In the following, we will discuss and describe the quality of these three emotional datasets.

### 2.1. EMO-DB

The Berlin Speech Emotion Database (EMO-DB) [27] was recorded at the Technical University, Berlin. It contains seven classes of basic emotions (Anger, Fear, Happiness, Disgust, Boredom, Sadness, and Neutral). Ten professional German actors (five men and five women) spoke ten sentences in German. Table 1 shows the ten different sentences in German. The 535 sentences were not equally distributed between the various emotional states. This distribution of emotional sentences for EMO-DB is shown in Table 2.

### 2.2. eNTERFACE Corpus

The eNTERFACE corpus is a further public, yet audio-visual emotion database. It consists of six emotional classes: Anger, Disgust, Fear, Happiness, Sadness, and Surprise [28]. The 42 subjects (eight women) from 14 nations were recorded in English in an office environment. Table 3 presents the selected emotional sentences. In addition, Table 4 shows the distribution of emotional sentences for eNTERFACE in which the 1277 sentences were not equally distributed between the various emotional states.

### 2.3. Self-Recording Database (KHUSC-EmoDB)

The recording of the corpus of KHUSC-EmoDB comprises Mandarin language sentences. Its contents were all produced by students from Shih-Chien University. The emotional voice of this corpus is recorded from four women and 13 men. Each speaker is recorded in all four emotions (Happiness, Fear, Sadness and Anger), so a total of 408 sentences for four emotions are presented in Table 5.

In order to guide the recording before the recording's emotional performance, a pre-process was done by viewing the corresponding emotional film or video content. In our experiment, seventeen normal recordings of a young person watching four different movies were made to register “Happiness”, “Fear”, “Sadness” and “Anger” and other emotions. The 408 sentences expressing the different emotional sentences were equally distributed among the various emotional states as shown in Table 6.

## 3. Proposed ESS System

**Figure 1.**
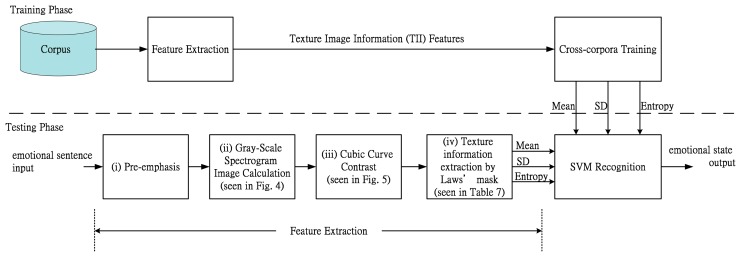
Diagram of the proposed TII-based ESS Algorithm.

Figure 1 shows a block diagram of the proposed ESS system using TII features. The feature extraction block computes the proposed TII features and the TII features are available to determine the emotional status of a speaker from his/her speech.

The proposed texture image informant (TII) features consists of four main parts: (i) pre-emphasis, (ii) gray-scale spectrogram image calculation, (iii) cubic curve contrast, and (iv) texture information extraction by Laws' mask. The classification block includes a SVM based classifier. SVM, a supervised learning algorithm, is usually used for classification and regression. It has been very popular in recent years due to its remarkable performance. In order to let the proposed TII-based ESS be insensitive to different languages, cross-corpus training will be required. The support vector machine (SVM) is used as classification tool with Laws' Mask (5 × 5) 42-dimentional feature vectors: Mean, SD and Entropy. The related-blocks of the proposed ESS system are then described in the following subsections.

### 3.1. Texture Image Information (TII) Features

In this section, a new method that extracts texture image features from speech spectrograms is presented. It is well known that a 2D narrowband speech spectrogram is a graphical display of the squared magnitude of the time-varying spectral characteristics of speech [29]. It is compact and highly efficient representation carrying information about parameters such as energy, pitch F0, formants and timing. These parameters are the acoustic features of speech most often used in emotion recognition systems [30–32]. The additional advantage is that by analyzing a speech spectrogram, it is more likely to preserve and take into account speech features that are caused by specific psycho-physiological effects appearing with certain emotions.

**Figure 2.**
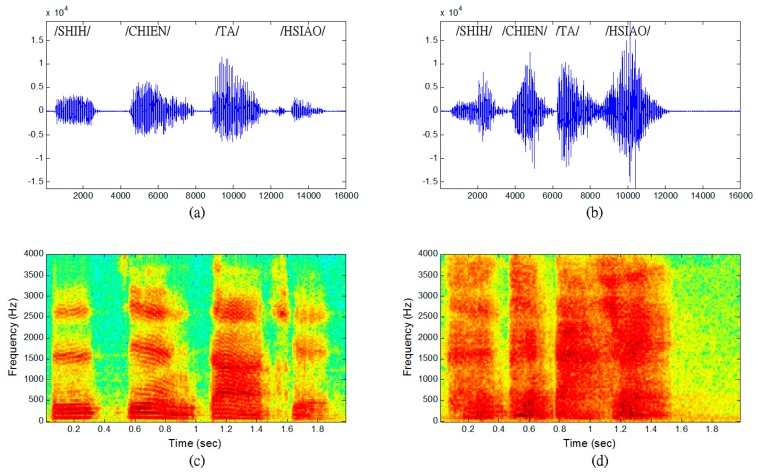
The input speech signals and the corresponding spectrograms. Speech uttered in Mandarin sentence “SHIH-CHIEN-TA-HSIAO.” (**a**) “Neutral” speech. (**b**) “Anger” speech. (**c**) Spectrogram of “Neutral” speech. (**d**) Spectrogram of “Anger” speech.

The input speech signals and the corresponding spectrograms for various emotion spectrograms used as an example are shown in Figure 2. The two speech signals pronounce in Mandarin the sentence “SHIH-CHIEN-TA-HSIAO”, one uttered with “Neutral” emotion and the other with “Anger” emotion. Some visible differences can be observed in terms of signal duration and amplitude in Figure 2a,b. The speech uttered with “Anger” emotion has less duration than that uttered with “Neutral” emotion. The average amplitude of the signal has a higher value in case of the speech signal uttered with “Anger” emotion. Compared to the speech uttered with “Neutral” emotion in Figure 2c,d, the spectrograms show that the frequencies have shifted upward or have higher values in the speech signal uttered with “Anger” emotion.

With increasing level of stress, the spectrograms revealed increasing formant energy in the higher frequency bands, as well as clearly increasing pitch for strong level stress. Other acoustic information, such as the formants also vary under different levels of stress. These observations indicate that the representation of texture image on spectrogram usually contains distinctive patterns that capture different characteristics of “Neutral” emotion and “Anger” emotion signals. Furthermore, the TII features in spectrograms can be used to discriminate the differences between various emotional levels in speech. In conclusion, the texture image information (TII) feature can provide the discrimination between different emotions in visual expression except for the conveyance pitch and formant tracks. In addition, the de-noising in images can be more easily completed than de-noising in speech signals.

### 3.2. Spectrogram Image Calculation

**Figure 3.**
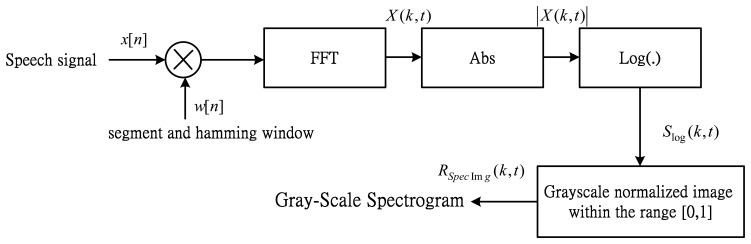
Extraction of the proposed TII features.

In this subsection, the 1-D speech signals are transformed into 2-D spectrogram images. Inspired by the concept of spectrogram image features [33], we generate the spectrogram images with time-frequency-intensity representation as shown in Figure 3.

First, the time-frequency-intensity representation, *X*(*k*,*t*), is determined by applying to the input signal with the windowed Short-Time Fourier Transform (STFT), which is given by:

(1) $$X(k,t)=\sum_{n=0}^{N-1}x[n]w[n-t]e^{-\frac{2\pi i}{N}kn},\;k=0,\ldots \ldots ,N-1$$

where *x*[*n*] is the input speech signal after pre-emphasis. *N* is the length of the window, *w*[*n*] is the Hamming window function and *k* corresponds to the frequency *f*(*k*) = *kf_s_*/*N*, where *f_s_* is the sampling frequency in Hertz.

Owing to the logarithmic of the human perception of sound, the log-spectrogram defined as:

(2) $$S_{\log}(k,t)=\log(\left|X(k,t)\right|)$$

Next, the spectrogram image representation, *R_SpecImg_* (*k*,*t*), is defined by the log-spectrogram is normalized into a grayscale normalized image, within the range from 0 to 1:

(3) RSpecImg(k,t)=Slog(k,t)−SminSmax−Smin

### 3.3. Image Contrast Enhancement Using Cubic Curve

**Figure 4.**
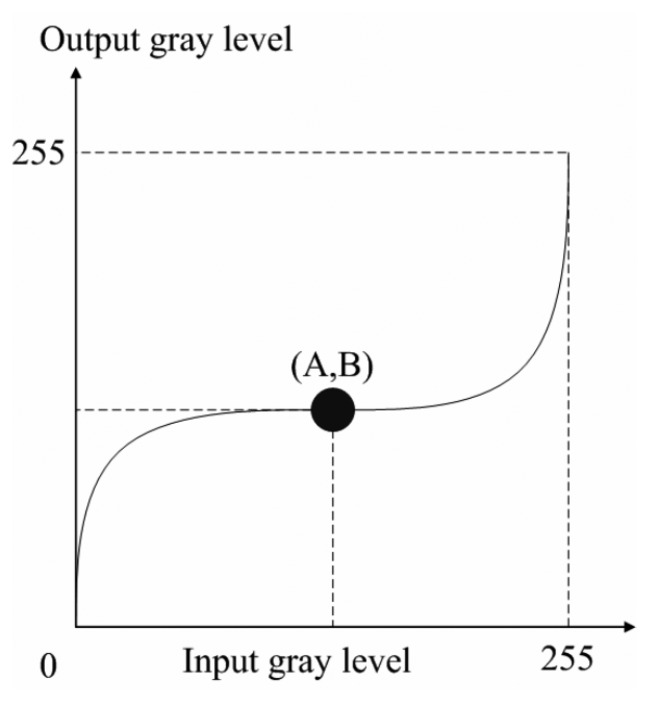
The different compensation curves based on different turning points.

The feature extraction task can be processed smoothly while upgrading the contrast of the gray-scale spectrogram images. A cubic curve is utilized to enhance the image to adjust its contrast [34]. Figure 4 shows the appropriate adjusting curve. It is observed that the adjusting curve contains an inflection point, so a variety of different curvatures of the curve can be produced by controlling the inflection point. Based on the above motivation, adjusting the curve inflection point to change the image contrast is utilized.

First, we assume that curve must pass through the two points (0,0) and (255,255), and the cubic curve is as in Equation (4) shown below:

(4) $$y=f(x)=ax^{3}+bx^{2}+cx+d$$

where *x* is the pixel value in the original image, *y* is the pixel value of the image after adjusting the curve. In Figure 4, “A” represents a cubic curve inflection point in the x-coordinate. In order to determine the unknown variables in all three curves, the study uses parameter “A” to obtain the needed compensation curve, wherein all variables in the cubic curve calculated by Equation (5) are replaced in Equation (8) below:

(5) $$A=\min_{x\in I}\left\{x\right\}+0.7\left\{\max_{x\in I}\left\{x\right\}-\min_{x\in I}\left\{x\right\}\right\}$$

(6) $$b^{2}=3\times a-{\left(255\right)}^{2}\times 3a^{2}-255\times 3\times a\times b$$

(7) $$a=\frac{1}{{\left(255\right)}^{2}-3\times 255\times A+3\times A^{2}}$$

(8) $$c=1-a\times {\left(255\right)}^{2}-b\times 255$$

where *I* is an image, *x* is the image pixel value at any point in the image. min*_x_*_∈_*_I_*{*x*} is represented as a minimum pixel value. min*_x_*_∈_*_I_*{*x*} is expressed as the maximum pixel value of the image.

**Figure 5.**
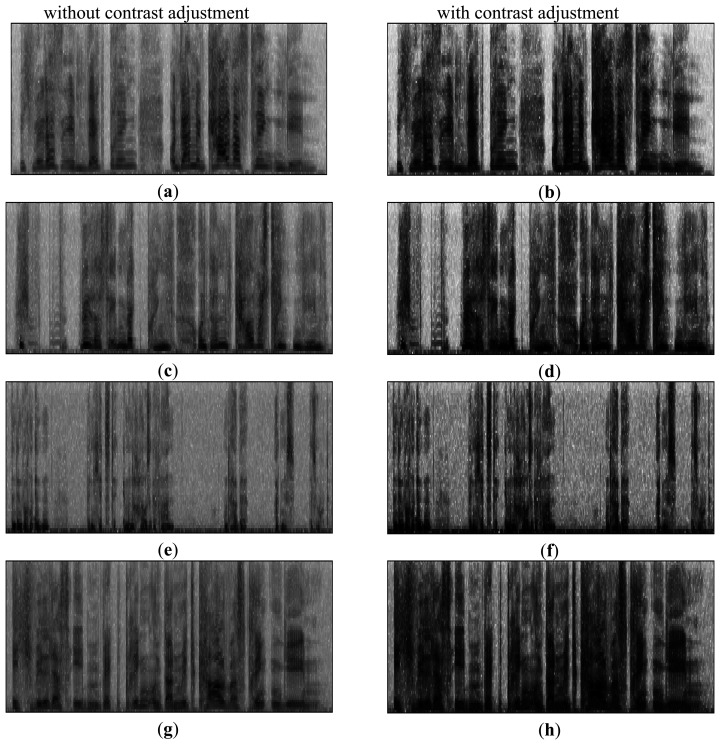
Spectrogram without/with contrast adjustment after the original image: (**a**) and (**b**) represent “Happiness”, (**c**) and (**d**) denote “Fear”, (**e**) and (**f**) are “Sadness”, (**g**) and (**h**) are “Angry”.

In general, the texture information is the main resonance frequency of the speech signal. After contrast adjustment, the size of each frequency component of spectrogram for the original speech sound is highlighted. Then, the image can obviously reflect the texture information in the spectrogram. Observing Figure 5, we can see the original “Happiness”, “Fear”, “Sadness” and “Angry” emotions in four spectrograms without/with contrast adjustment. The performances show different texture image information in the spectrograms. After compensating by image contrast, each texture image information for each emotion can effectively displayed and discriminated.

### 3.4. Extraction of Texture Image Information by Laws' Masks

The TII features derived from spectrogram images will be extracted by the Laws' masks based on the principle of texture energy measurement [35]. The Laws' masks are well described for texture energy variation in image processing. In general, the Laws' masks consist of five masks derived from 1-D vectors, such as edge (*E*_5_), level (*L*_5_), spot (*S*_5_), ripple (*R*_5_) and wave (*W*_5_). All the masks were expressed in the Equations (9)–(13):

(9) $$E_{5}=\text{Edge detection}:[-1\;-2\;0\;2\;1]$$

(10) $$L_{5}=\text{Level detection}:[1\;4\;6\;4\;1]$$

(11) $$S_{5}=\text{Spot detection}:[-1\;0\;2\;0\;-1]$$

(12) $$R_{5}=\text{Ripple detection}:[1\;-4\;6\;-4\;1]$$

(13) $$W_{5}=\text{Wave detection}:[-1\;2\;0\;-2\;1]$$

2-D filters of size 5 × 5 were generated by convoluting any vertical 1-D vector with a horizontal one. Finally, the 25 combinations of 2-D mask are shown in Table 7.

First, we convoluted the image with each 2-D mask to extract texture information from an image Im_(_*_i_*_,_*_j_*_)_ of size (*M* × *N*). For example, The *M_E_*_5_*_E_*_5_ is used to filter the image Im_(_*_i_*_,_*_j_*_)_, we can see the regarded as “texture image” (TIm*_E_*_5_*_E_*_5_) shown in the Equation (14):

(14) TImE5E5=Im(i,j)⊗ME5E5

All the 2-D masks, except *M_L_*_5_*_L_*_5_, had zero mean. According to Laws, the normalization Norm(TIM*_mask_*) can be determined while the contrast of all the texture images TIM_(_*_i_*_,_*_j_*_)_ is normalized by the texture image TIm*_L_*_5_*_L_*_5_ shown in the Equation (15):

(15) $$\text{Norm}({\text{TIm}}_{\text{mask}})={\text{TIm}}_{\text{mask}}/{\text{TIm}}_{L_{5}L_{5}}$$

Next, we can calculate a non-linear interval by processing a normalized TIm and yield through a “Texture Energy Measurements, (TEM)” filter. These consist of a moving non-linear window average of the absolute values in Equation (16):

(16) $${\text{TEM}}_{\left(i,j\right)}=\sum_{u=-7}^{7}\sum_{v=-7}^{7}\left[\text{Norm}({\text{TIm}}_{i+u,j+v})\right]$$

However, not all mask energy can be used as the input basis of texture energy. Hence, we rotate the values within 25 masks from yielded TEM, and take out unchangeable 14 Rotationally Invariant Texture Energy Measurements (noted RITEM) values before and after rotation. The RITEM represents the calculation value of Laws' Mask, which are the values specially used to measure the texture energy as seen in the Equation (17):

(17) $${\text{RITEM}}_{E5L5}=\left({\text{TEM}}_{E5L5}+{\text{TEM}}_{L5E5}\right)/2$$

After Equation (17), we use this results to extract three texture feature values: Mean, Standard Deviance (SD) and Entropy in Equations (18)–(20). These three features are used to judge the variation of texture information. Equation (18) to Equation (20) are the calculation formulae of three features values, where TR_(_*_i_*_,_*_j_*_)_ represents the unchangeable values within 25 masks from TEM before and after rotation, *M* × *N* represents the size of whole image. Finally, each equation will have 14-dimensional feature vectors. A total of three feature vectors are 42-dimensional and the feature vectors will be used as the input for training the SVM classifier:

(18) $$\text{Mean}=\frac{\sum_{i=0}^{M}\sum_{j=0}^{N}\left[{\text{RITEM}}_{\left(i,j\right)}\right]}{M\times N}$$

(19) $$\text{SD}=\sqrt{\frac{\sum_{i=0}^{M}\sum_{j=0}^{N}{\left({\text{RITEM}}_{\left(i,j\right)}-\text{Mean}\right)}^{2}}{M\times N}}$$

(20) $$\text{Entropy}=\frac{\sum_{i=0}^{M}\sum_{j=0}^{N}{\left({\text{RITEM}}_{\left(i,j\right)}\right)}^{2}}{M\times N}$$

### 3.5. Support Vector Machine (SVM)

After extracting the texture image information, the next stage is emotional state classification. The support vector machine (SVM), a supervised learning algorithm, is usually used for classification and regression. It has been very popular in recent years due to its remarkable performance. In this paper, we adopt the SVM as our emotion classifier. The SVM needs to be given a set of samples belonging to two classes for the training phase. To completely distinguish these two classes [36], we need to find a hyper-plane. We have the training data set: {*I_n_*,*T_n_*}, where *n* = 1,2,…,*N* and *I_n_* is the n-dimensional input feature vector. *T_n_*_∈_ {1,−1} is target output of emotional class labeled. The training data is used to find the best hyper-plane, and is used to classify the data. Then, the decision function is given as follows:

(21) $$D_{f}(\text{I},\text{w},c)=\mathrm{sgn}(\text{I}\cdot \text{w}+c)$$

Considering the optimization problem, the optimal separating hyper-plane can be determined by minimizing *ϕ*(w)=‖w‖^2^/2 as:

(22) $$T_{n}(\text{w}\cdot {\text{I}}_{n}+c)\geq 1,n=1,2,\ldots ,N$$

By introducing Lagrange multipliers α, the constrained problem becomes:

(23) $$\min\varphi (\text{w},\Xi )=\frac{1}{2}{\left\|\text{w}\right\|}^{2}+d(\sum_{n=1}^{N}\xi_{n})$$

Then, we employ a kernel function *k*(*I*,*I_n_*), the decision function for final hyper-plane is shown as below:

(24) $$D_{f}(\text{I})=\mathrm{sgn}\left(\sum_{n=1}^{N}\alpha_{n}T_{n}k(\text{I},{\text{I}}_{n})+c\right)$$

**Figure 6.**
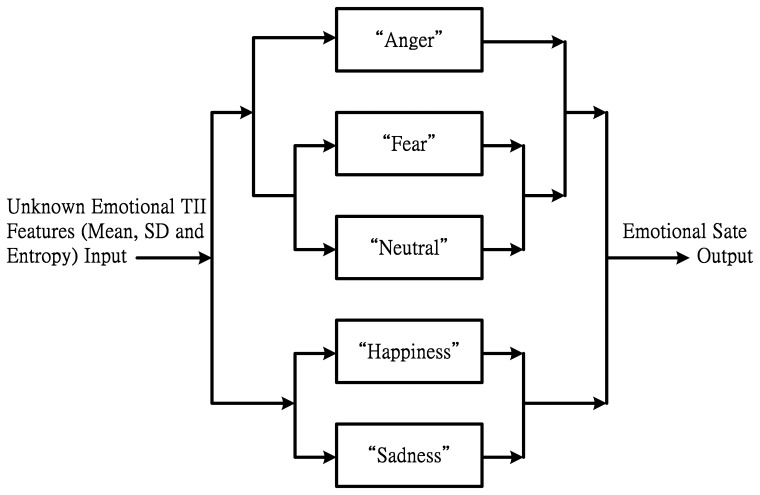
The classification decision applying in SVM.

Figure 6 shows the classification decision of SVM recognition. The compared sequence and scheduling method is based on the recognition results of SVM. We can see that the emotional state with higher recognition rate is compared *a priori* with other emotional states. First, the unknown emotional TII feature input via classification decision is “Happiness” or “Sadness”. At the same time, “Fear” is also a classification decision or “Neutral”. Next, assuming that the recognition results are “Happiness” and “Fear”, and then the “Neutral” and “Angry” can judge each other. Finally, the classification result is then compared with “Angry” to determine which emotion state it really is.

## 4. Experiments and Results

In our experimental results, we use three types of corpora to compare and demonstrate the proposed TII-based emotion sensing system for recognizing emotional states. The corpus consists of three corpora: EMO-DB, eNTERFACE, and KHUSC-EmoDB and has been described in Tables 1 and 6. The three corpora are split into two to form the training and testing sets. In order to reasonably evaluate the performance of the proposed TII-based feature extraction in each corpus, this paper uses a common emotional class among the corpus for testing sets. In addition, the SVM classifier is trained on the training set. First, the proposed TII-based ESS system are evaluated under the extracted four common emotional class labels: “Happiness”, “Fear”, “Sadness” and “Anger” from the three corpora. Second, the performance of the proposed TII-based algorithm will be evaluated with confusion matrix of the methodology between emotions for all speakers.

### 4.1. The Corpora

In this subsection, the common emotional states from three corpora: EMO-DB, eNTERFACE, and KHUSC-EmoDB are extracted. There are four types of emotional class labels: “Happiness”, “Fear”, “Sadness” and “Anger” in Table 8. In order to further evaluate the cross-corpus performance, the row labeled as “Mixed” is used to represent a mix of the three abovementioned corpora. The total of the mixed corpora is 1584 sentences.

### 4.2. Confusion Matrix

Tables 9 and 12 are the confusion matrices, which are widely used graphical tools that reflect the performance of an algorithm. Each row of the matrix represents the instances of a predicted class, while each column represents the instances of an original class. Thus, it is easy to visualize the classifier's errors while trying to accurately predict each original class' instances. Moreover, the original classes listed in the second row are the four emotional classes. The rows 3–7 in Tables 10 and 11 and rows in Table 12 are used to record the amount (percentage) of the test data being classified to the original emotion. Finally, the last row of each table is the average recognition accuracy of the proposed ESS with TII features for each corpus.

### 4.3. Evaluation of Contrast Adjustment without/with Cubic Curve

The comparison between without/with cubic curve, which is used for image enhancement, will be evaluated in this subsection. In fact, the spectrogram of the original image contains many non-voiced parts of the information on pronunciation. After contrast adjustment with the cubic curve, we can efficiently enhance non-voiced pronunciation in the spectrogram images, so the intensity variation for the emotional status of speaker pronunciation can be presented in detail.

**Figure 7.**
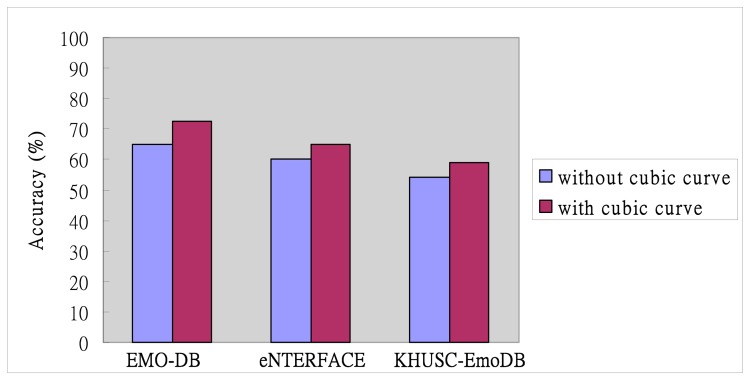
Recognition accuracy without/with cubic curve among the three corpora.

Figure 7 shows the emotion recognition accuracy without/with cubic curve among the three corpora: EMO-DB, eNTERFACE, and KHUSC-EmoDB. For the EMO-DB, the average rate for correct emotional recognition is about 65%. In contrast, the evaluation of contrast adjustment with cubic curve can achieve an average rate 72.5% for correct emotional recognition. In the eNTERFACE corpus, the contrast adjustment without/with cubic curve also achieves 60% and 65%, respectively. For KHUSC-EmoDB, the evaluation result with cubic curve (59%) is better than the result without cubic curve (54%). In conclusion, it is observed that the recognition accuracy performed on EMO-DB is better than in the other two corpora. Based on the above experiments, we can understand the contrast adjustment with cubic curve is helpful for the proposed EES system. Therefore, the next evaluations will utilize the method of contrast adjustment with cubic curve to perform the all experiments.

### 4.4. Evaluation of the Proposed TII-Based ESS Using SVM Classifier on Three Corpora

In Table 8, we have found that there are different amounts of examples of each emotional state in each speech database. To be fair in it evaluation of the various emotional recognition rates, our experiments use a minimum number of emotional class in each corpus as a test standard. The training set and testing set are not overlapped in order to achieve an open test. For example, 62 sentences is the minimum among the four kinds of emotion. We use 62 sentences as the number of each emotional test on the EMO-DB speech database. Then, the test set and training set number is 31, respectively. In addition, 207 sentences is the minimum for the “Happiness” emotion for the eNTERFACE speech database. We use 103 sentences as test set and 104 sentences as training set, respectively. Because 102 sentences is the same number for each emotional state in the KHUSC-EmoDB database, the testing set and training set are both 51 sentences, respectively.

**Figure 8.**
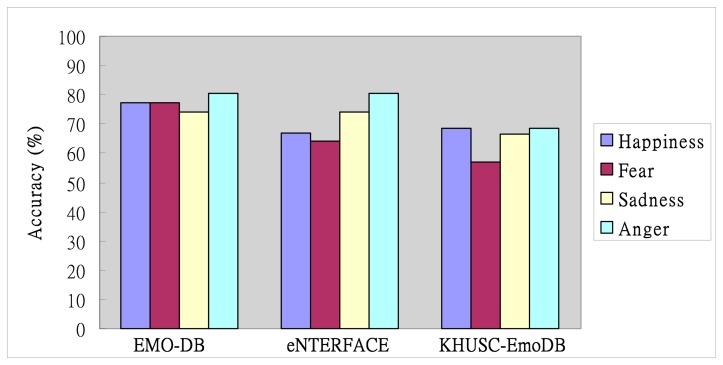
Evaluation of TII-based ESS on EMO-DB, eNTERFACE and KHUSC-EmoDB.

In Figure 8, the evaluation of TII-based ESS using SVM on EMO-DB shows that the recognition accuracy of the “Anger” emotion can achieve 80.65% and outperform other emotional recognition accuracies. Furthermore, the evaluation of TII-based ESS using SVM for four-class task can achieve 77.42% average accuracy of emotion recognition in the EMO-DB. In other words, the figure shows that the recognition accuracy of “Anger” emotion also can achieve 80.65%. The recognition accuracies of “Happiness” and “Fear”, however, are lower than for other emotional states. The average accuracy on eNTERFACE is about 73.06%, and it is lower than that on EMO-DB. In addition, the evaluation results reveal that the proposed TII-based ESS using SVM can model the emotional states in the KHUSC-EmoDB except for the “Fear” emotional classification. However, the performance on KHUSC-EmoDB is also lower than that on EMO-DB. For the comparison among the three corpora, the evaluation results on EMO-DB outperform the other two corpora. It is found that the proposed TII-based ESS is suitable to apply on the EMO-DB.

### 4.5. Evaluation of on Cross-Corpora for Training/Testing

In order to let the proposed TII-based ESS be insensitive to different language, cross-corpus training will be required. The support vector machine (SVM) is used as classification tool with Laws' Mask (5 × 5) 42-dimentional feature vectors: Mean, SD and Entropy.

**Figure 9.**
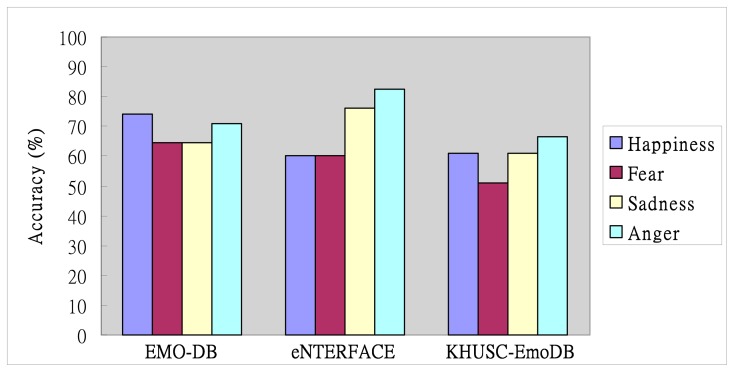
Evaluation of mixed corpora for training and three corpora for testing.

Figure 9 shows the results of the proposed TII-based ESS with a mixed database set for cross-corpora training/testing. This figure shows the confusion matrix of the results of the average accuracy of the proposed approach on the three corpora for testing and mixed corpora for training. In EMO-DB, the lowest accuracy of emotional recognition is 64.52% in “Fear”, and it is same as that in “Sadness”. Finally, the average accuracy of the proposed approach for testing EMO-DB is 68.55%. In eNTERFACE, the figure also shows that “Happiness” and “Fear” are both classified with 60.19% accuracy. The average accuracy with 69.78% has been improved against other two corpora for testing. In addition, the evaluation results of the average accuracy of the proposed approach on KHUSC-EmoDB for testing. We can find that the emotional recognition accuracy in “Fear” is 50.98% and is the lowest among all the emotional states. The average accuracy is 59.8%. For the comparison between the two corpora KHUSC-EmoDB and EMO-DB, the evaluation results of the proposed ESS under KHUSC-EmoDB are lower than the results under EMO-DB. Observing the Figure, we can conclude that the performance of the proposed ESS for KHUSC-EmoDB is lower than that for other two corpora. In addition, experimental results show that the accuracy for the emotional state “Fear” is the lowest among all the emotional states.

### 4.6. Comparison with the Existing Method

In this subsection, a comparison of the proposed TII-based ESS with existing techniques will be presented. Table 9 shows a comparison between the proposed TII feature set with SVM classifier and the existing algorithms that use standard MFCC features with the HMM/SVM classifiers in Ashish's work [37] and using brute-force features with SMO classifier in Escalona Mena's. Observing Table 9, it is found that we have a five-class task for Ashish and a seven-class task for Escalona Mena while the proposed TII-based ESS system is a four-class task. In addition, the feature sets and classifier are also different among the three ESS systems.

The evaluation of ESS on EMO-DB with the Ashish method using MFCC features with HMM classifier is shown in Table 10. Results show that high accuracy is observed in the classification of “Anger”. The average of recognition accuracy is 69.88%. However, the average recognition rate for the three common emotional labels “Anger”, “Happiness” and “Sadness” (called AHS Accuracy) is 67.66%.

Table 11 presents that the evaluation of ESS on EMO-DB proposed by Ashish using the MFCC features with SVM classifier. We also find that the classification of “Happiness” is the worst result. The average accuracy can only achieve a performance of 67.66%. In AHS accuracy, the results also get only 66.66%.

Table 12 shows the evaluation of the ESS system using the brute-force feature with SMO classifier. We also find that the classification of “Anger” is the best result. The average accuracy can achieve a high performance of 82.50%. Furthermore, the results can achieve 83.33% in AHS accuracy.

Table 13 shows the comparison evaluation of the ESS system with the corresponding feature extraction. The performance of brute-force feature extraction with SMO has proven to be a very good presentation. Based on the findings, the brute-force feature extraction is then utilized in the proposed TII-based ESS system with SVM classifier. It is found that the two feature extractions can compensate for each other when low accuracy of some emotional state occurs. For example, the accuracy of “Happiness” emotion can be improved from 70.00% to 78.80%. Furthermore, the AHS Accuracy has increased from 83.33% to 85.88%.

Table 14 shows the computational time for extraction of the various feature sets. The computation time is evaluated under a Windows platform using an Intel Duo-core processor at 2.26 GHz. Although the average accuracy for brute-force extraction of acoustic parameters is obviously higher than that for the proposed TII-based feature extraction, it needs more computational time to maintain its high performance. Considering the efficiency, the proposed TII-based feature extraction on ESS system is superior to the brute-force feature extraction on the ESS system.

## 5. Discussion and Conclusions

In this paper, a novel feature extraction based on texture image information (TII) features for emotion sensing in speech is presented. The TII features are shown to be a reliable source for emotion feature extraction, which is known to give more time-frequency-intensity representation. The experimental evaluations on the three emotional datasets: EMO-DB, eNTERFACE and KHUSC-EmoDB show statistically significant performance improvements with the TII features as compared to the MFCC features.

First, we also performed the evaluation of contrast adjustment with/without cubic curve. The evaluation reveals that the cubic curve can enhance the texture information. Second, the three texture feature sets: Mean, Standard Deviance and Entropy successfully describe the discrimination between various emotion states. We can show that the TII feature set can more successfully identify emotion status than the other conventional features through spectrogram image calculation and Laws' masks. Next, the LLD and functional for the large scale brute-force feature sets are utilized with the proposed TII features. In order to evaluate the large scale brute-force feature sets associated with the proposed TII feature set, the robustness of ESS with various languages has been tested. Our experiment results also show that the TII feature set with the large scale brute-force feature sets can improve the robustness of ESS under various languages. Although the average accuracy for brute-force extraction of acoustic parameters is obviously higher than that for the proposed TII-based feature extraction, it needs more computational time to ensure the high performance. Considering the efficiency, the proposed TII-based feature extraction on ESS system is superior to the brute-force feature extraction on ESS systems. In comparison with the existing ESS algorithm, we also find that the proposed feature set is excellent for distinguishing emotion, and superior to other method using 1-D MFCC. Experimental results show that the correct classification rates range from 65.20% to 77.42% for different language databases. In our future work, a strategy for multi-resolution will be integrated into the TII-based feature extractor, resulting in a high accuracy performance for an ESS system.

## Figures and Tables

**Table 1.** The emotional sentences for EMO-DB.

EMO-DB	

Rank	Sentence
1	Der Lappen liegt auf dem Eisschrank.
2	Das will sie am Mittwoch abgeben.
3	Heute abend konnte ich es ihm sagen.
4	Das schwarze Blatt Papier befindet sich da oben neben dem Holzstuck.
5	In sieben Stunden wird es soweit sein.
6	Was sind denn das fur Tuten, die da unter dem Tisch stehen.
7	Sie haben es gerade hochgetragen und jetzt gehen sie wieder runter.
8	An den Wochenenden bin ich jetzt immer nach Hause gefahren und habe Agnes besucht.
9	Ich will das eben wegbringen und dann mit Karl was trinken gehen.
10	Die wird auf dem Platz sein, wo wir sie immer hinlegen.

**Table 2.** Distribution of emotional sentences for EMO-DB.

EMO-DB	**Anger**	**Boredom**	**Disgust**	**Fear**	**Happiness**	**Sadness**	**Neural**	***Total***

	127	81	46	69	71	62	79	***535***

**Table 3.** The emotional sentences for eNTERFACE.

eNTERFACE Database	

Rank	Sentences
1	What??? No, no, no, listen! I need this money!
2	I don't care about your coffee! Please serve me!
3	I can have you fired you know!
4	Is your coffee more important than my money?
5	You're getting paid to work, not drink coffee!
6	Life won't be the same now
7	Oh no, tell me this is not true, please!
8	Everything was so perfect! I just don't understand!
9	I still loved him (her)
10	He (she) was my life
11	That's great, I'm rich now!!!
12	I won: this is great! I'm so happy!!
13	Wahoo… This is so great.
14	I'm so lucky!
15	I'm so excited!
16	Oh my god, there is someone in the house!
17	Someone is climbing up the stairs
18	Please don't kill me…
19	I'm not alone! Go away!
20	I have nothing to give you! Please don't hurt me!

**Table 4.** Distribution of emotional sentences for eNTERFACE.

eNTERFACE	**Anger**	**Disgust**	**Fear**	**Happiness**	**Sadness**	**Suprise**	***Total***

	215	215	215	207	210	215	***1277***

**Table 5.** The emotional sentences for KHUSC-EmoDB.

KHUSC-EmoDB Database	

Rank	Sentence
1	怎麼會這樣(zen3 me0 hui4 zhe4 yang4)
2	你在哪?(ni3 zai4 na3)
3	這是我的書(zhe4 shi4 wo3 de0 shu1)
4	真沒想到你會這樣(zhen1 mei2 xiang3 dao4 ni3 hui4 zhe4 yang4)
5	我做了一個夢(wo3 zuo4 le0 yi1 ge0 meng4)
6	他知道這件事了(ta1 zhi1 dao4 zhe4 jian4 shi4 le0)

**Table 6.** Distribution of emotion for KHUSC-EmoDB.

KHUSC-EmoDB	**Anger**	**Happiness**	**Sadness**	**Fear**	***Total***

	102	102	102	102	***408***

**Table 7.** The result list for mutual combinations of 2-D laws' masks.

**L_5_^T^L_5_**	**E_5_^T^L_5_**	**S_5_^T^L_5_**	**W_5_^T^L_5_**	**R_5_^T^L_5_**
**E_5_^T^L_5_**	**E_5_^T^E_5_**	**S_5_^T^E_5_**	**W_5_^T^E_5_**	**R_5_^T^E_5_**
**S_5_^T^L_5_**	**E_5_^T^S_5_**	**S_5_^T^S_5_**	**W_5_^T^S_5_**	**R_5_^T^S_5_**
**W_5_^T^L_5_**	**E_5_^T^W_5_**	**S_5_^T^W_5_**	**W_5_^T^W_5_**	**R_5_^T^W_5_**
**R_5_^T^L_5_**	**E_5_^T^R_5_**	**S_5_^T^R_5_**	**W_5_^T^R_5_**	**R_5_^T^R_5_**

**Table 8.** Description of the collected speech database.

**Emotional State**	**Happiness**	**Fear**	**Sadness**	**Anger**	***Total***

**Corpora**					
**EMO-DB**	71	69	62	127	***329***
**eNTERFACE**	207	215	210	215	***847***
**KHUSC-EmoDB**	102	102	102	102	***408***
**Mixed**	***380***	***386***	***374***	***444***	***1584***

**Table 9.** A comparison between the proposed method and some existing methods.

Author	Corpus	Emotional Class	Feature Set	Classifier
The proposed	EMO-DB	Happiness Fear Sadness Anger	TII (42-dimentional feature vectors)	SVM
Ashish [37]	EMO-DB	Happiness Sadness Anger Surprise neutral	MFCC (39-dimentional feature vectors)	HMM SVM
Escalona Mena [26]	EMO-DB	Anger Boredom Disgust Fear-Anxiety Happiness Neural	brute-force feature extraction (LLD and functionals)	SMO

**Table 10.** Evaluation of ESS proposed by Ashish using MFCC feature with HMM classifier.

Corpus	Original Emotion	Recognition Rate (%)				
	Predicted Emotion	Anger	Happiness	Sadness	Surprise	Neutral
Emo-DB	Anger	**83.33%**	16.67%	0.00%	0.00%	0.00%
	Happiness	0.00%	**57.14%**	14.29%	28.57%	0.00%
	Sadness	0.00%	0.00%	**62.50%**	12.50%	25.00%
	Surprise	28.57%	0.00%	0.00%	**71.43%**	0.00%
	Neutral	0.00%	0.00%	25.00%	0.00%	**75.00%**
**Average Accuracy (%)**		**69.88%**				
**AHS Accuracy (%)**		**67.66%**				

**Table 11.** Evaluation of ESS proposed by Ashish using MFCC feature with SVM classifier.

Corpus	Original Emotion	Recognition Rate (%)				
	Predicted Emotion	Anger	Happiness	Sadness	Surprise	Neutral
**EMO-DB**	Anger	**71.42%**	14.29%	0.00%	14.29%	0.00%
	Happiness	0.00%	**57.14%**	14.29%	28.57%	0.00%
	Sadness	0.00%	0.00%	**71.43%**	0.00%	28.57%
	Surprise	22.39%	14.28%	0.00%	**63.33%**	0.00%
	Neutral	0.00%	0.00%	25.00%	0.00%	**75.00%**
**Average Accuracy (%)**		**67.66%**				
**AHS Accuracy (%)**		**66.66%**				

**Table 12.** Evaluation of ESS proposed by Escalona Mena using brute-force feature with SMO classifier.

Corpus	Original Emotion	Recognition Rate (%)						
	Predicted Emotion	Anger	Boredom	Disgust	Fear-Anxiety	Happiness	Neural	Sadness
**EMO-DB**	Anger	**90.00%**	0.00%	2.50%	2.50%	5.00%	0.00%	0.00%
	Boredom	0.00%	**75.00%**	0.00%	0.00%	0.00%	75.00%	7.50%
	Disgust	2.50%	2.50%	**87.50%**	2.50%	0.00%	2.50%	2.50%
	Fear-Anxiety	5.00%	0.00%	2.50%	**85.00%**	7.50%	0.00%	0.00%
	Happiness	12.50%	0.00%	2.50%	15.00%	**70.00%**	0.00%	0.00%
	Neural	0.00%	15.00%	2.50%	2.50%	0.00%	**80.00%**	0.00%
	Sadness	0.00%	10.00%	0.00%	0.00%	0.00%	0.00%	**90.00%**
**Average Accuracy (%)**		**82.50%**						
**AHS Accuracy (%)**		**83.33%**						

**Table 13.** The comparison evaluation of ESS for three common emotional states.

Emotional States	Anger	Happiness	Sadness	*AHS* Accuracy
Methods				
TII-based feature extraction with SVM	80.65%	77.42%	74.19%	***77.42*%**
brute-force feature extraction with SMO	90.00%	70.00%	90.00%	***83.33*%**
MFCC feature extraction with HMM	83.33%	57.14%	62.50%	***67.66*%**
MFCC feature extraction with SVM	71.42%	57.14%	74.43%	***66.66*%**
TII + brute-force feature extraction with SVM	90.50%	78.80%	88.34%	***85.88*%**

**Table 14.** The computational time with/without brute-force feature set on the ESS system.

Requirement	Computational Times (sec)
Feature Set	
TII-based feature extraction with SVM	2.1
brute-force feature extraction with SVM	3.5
TII + brute-force feature extraction with SVM	3.8
